# Loss of lamin A function increases chromatin dynamics in the nuclear interior

**DOI:** 10.1038/ncomms9044

**Published:** 2015-08-24

**Authors:** I. Bronshtein, E. Kepten, I. Kanter, S. Berezin, M. Lindner, Abena B. Redwood, S Mai, S. Gonzalo, R. Foisner, Y. Shav-Tal, Y. Garini

**Affiliations:** 1 Physics Department and Nanotechnology Institute, Bar Ilan University, Ramat Gan 5290002, Israel; 2 The Mina & Everard Goodman Faculty of Life Sciences and Nanotechnology Institute, Bar Ilan University, Ramat Gan 5290002, Israel; 3 Edward A. Doisy Department of Biochemistry and Molecular Biology, School of Medicine, St Louis University, 1100 South Grand Ave. St Louis, Missouri 63104, USA; 4 Manitoba Institute of Cell Biology, Department of Physiology and Pathophysiology, University of Manitoba, Cancer Care Manitoba, Winnipeg, Manitoba, Canada R3E 0V9; 5 Max F. Perutz Laboratories, Medical University Vienna, Vienna Biocenter (VBC), Dr. Bohr-Gasse 9, 1030 Vienna, Austria

## Abstract

Chromatin is organized in a highly ordered yet dynamic manner in the cell nucleus, but the principles governing this organization remain unclear. Similarly, it is unknown whether, and how, various proteins regulate chromatin motion and as a result influence nuclear organization. Here by studying the dynamics of different genomic regions in the nucleus of live cells, we show that the genome has highly constrained dynamics. Interestingly, depletion of lamin A strikingly alters genome dynamics, inducing a dramatic transition from slow anomalous diffusion to fast and normal diffusion. In contrast, depletion of LAP2α, a protein that interacts with lamin A and chromatin, has no such effect on genome dynamics. We speculate that chromosomal inter-chain interactions formed by lamin A throughout the nucleus contribute to chromatin dynamics, and suggest that the molecular regulation of chromatin diffusion by lamin A in the nuclear interior is critical for the maintenance of genome organization.

## Abstract (web summary)

Nuclear lamins mediate interactions between chromatin and the nuclear envelope, however they are also found throughout the nucleoplasm. By measuring the dynamics of different genomic loci, Bronshtein *et al.* show that lamin A is also required for the stability of the nuclear interior.

## Graphical abstract

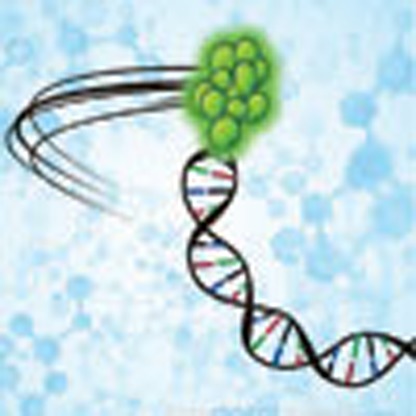

The cell nucleus is highly ordered at different levels, from the compaction of DNA into nucleosomes, to the complex compartmentalization of the genome into chromosomal territories^1^, which are organized in a compact unknotted state. The organization and compartmentalization of the genome in the three-dimensional (3D) nuclear space is crucial for proper cellular function^2^,^3^,^4^. Different models have been proposed to explain genome organization, including polymer models^5^,^6^, and structural models of chromatin anchorage to stable structures^7^. Polymer models^5^,^8^,^9^ are mainly based on interaction maps of genome loci measured by chromosome conformation capture techniques^10^. Nevertheless, the broad experimental variability of the polymer structure provided for different cells nuclei does not allow to establish a solid model for chromatin organization. Other studies show that specific chromosomal domains are anchored to the lamina^7^, a scaffold structure at the nuclear envelope. Some studies suggest a ‘nuclear matrix' that forms a rather stable structure throughout the nucleus that can support the chromosome structure^11^,^12^.

To gain further insight into genome organization within the nucleus, we focused on the effect of lamin A on the dynamic properties of different genomic regions in live cells. Together with B-type lamins, the A-type lamins, lamin A and C, form the nuclear lamina in most somatic mammalian cells. The lamina contributes to peripheral heterochromatin association and to nuclear integrity^13^, and its deficiency has severe effects on nuclear plasticity^14^ and chromatin organization^15^,^16^,^17^. Importantly, significant levels of A-type lamins are also found throughout the nucleoplasm, where their exact role remains unknown^13^. Lamin A and lamin B1 behave differently during post-mitotic nuclear assembly. Lamin B1 assembles around chromatin and localizes mainly at the nuclear periphery, while lamin A in early G1 initially localizes throughout the nucleus in a highly mobile pool, followed by a migration of a large fraction that assembles at the peripheral lamina^18^.

Dynamic studies of the nucleus were performed previously by using a variety of methods, either by following tagged proteins in the nucleoplasm or by tagging specific genomic sites and using a variety of imaging methods^15^,^19^, but it remained unclear how chromatin is dynamically organized in the nuclear interior and which components are involved. Chromatin dynamics are important for many processes in the nucleus, including gene regulation and the maintenance of genomic stability^20^.

To explore the organizational mechanisms of the genome in the nucleus, we studied the dynamics of different genomic regions in the nucleus of live cells, repeating the measurement in different cell lines and different genomic loci. We show that the depletion of lamin A strikingly alters genome dynamics, inducing a dramatic transition from slow anomalous diffusion to fast and normal diffusion. Rescuing lamin A in depleted cells fully recover the slow dynamics, but mutated lamin A only partially recovers the slow dynamics. Further, continuous photobleaching (CP) experiments show that ∼40% of lamin A is localized and bound throughout the nucleus. The results indicate that chromatin organization is actively controlled by chromosomal inter-chain interactions formed by lamin A throughout the whole nucleus and not only at the lamina. The suggested model provides a mechanism that can maintain genome organization.

## Results

### Live cell imaging of telomeres in the nucleus

**Figure 1:**
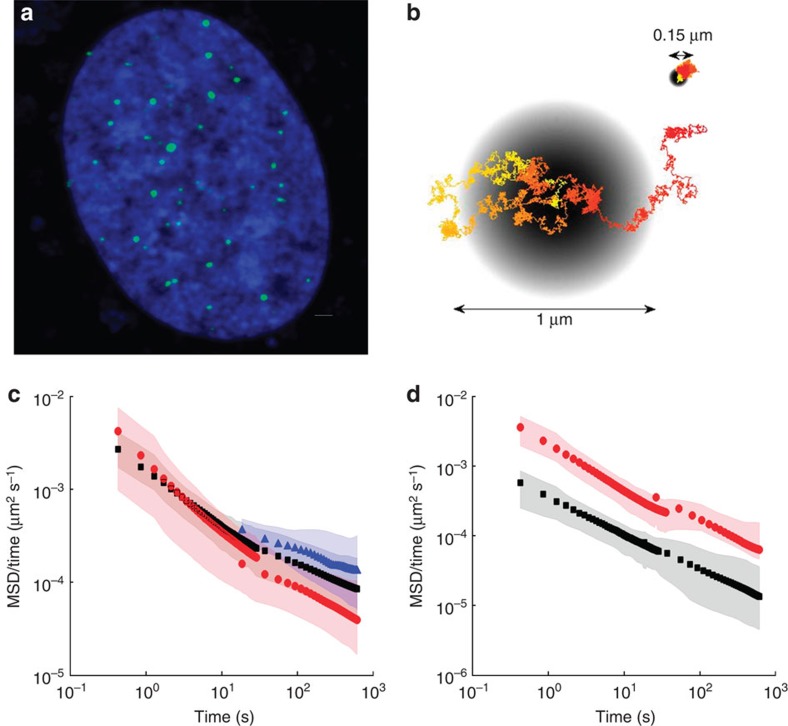
Chromatin diffusion is anomalous. (**a**) Fluorescence image of a typical nucleus expressing GFP–TRF2 proteins marking telomeres (green) with Hoechst staining (blue). Scale bar, 1 μm. (**b**) Gray circles show the MSD of an ensemble of simulated diffusing particles for normal diffusion (centre) with *α*=1, *D*=2.8 × 10^−4^ μm^2^ s^−1^ compared with anomalous diffusion with *α*=0.5, *D*=2.8 × 10^−4^ μm^2^ s^−0.5^ (top right). Grey levels indicate time, with black at *τ*=0 and lightest gray at *τ*=1,800 s. Overlaid are two representative trajectories, the colour represents time. The restricted nature of anomalous diffusion is clearly seen. (**c**) MSD divided by time in U2OS cells as measured for telomeres (black squares, *N*=958), centromeres (red circles, *N*=957) and a genomic locus consisting of an integrated *lacO* array (blue triangles, *N*=20). (**d**) MSD divided by time for telomere diffusion in NIH3T3 (black squares, *N*=325) and HeLa cells (red circles, *N*=166), all showing anomalous subdiffusion. Symbols designate the average locus MSD while shaded areas mark the s.d. of all single loci MSDs. Individual cell types are presented in Supplementary Figs 1 and 3.

To address these questions, we analysed the movement of fluorescently tagged genomic regions in living cells. Figure 1a shows a typical fluorescence image of telomeres labelled with green fluorescent protein (GFP)–TRF2 in live mouse embryonic fibroblasts (MEF) (Supplementary Fig. 1). The cells were imaged by confocal microscopy and individual trajectories **r**(*τ*) of labelled chromatin loci were extracted through single particle tracking (SPT) techniques^19^,^21^ (Fig. 1b, Supplementary Movie 1). For each trajectory, the time-averaged mean square displacement (MSD), 〈**r**^2^(*τ*)〉, was calculated with *τ* designating a time-lag along the trajectory.

Diffusion can be characterized as either normal or anomalous diffusion. In normal diffusion, such as Brownian motion, the MSD is linear in time 〈**r**^2^(*τ*)〉=2*dDτ*, where *D* is the diffusion constant and *d* is the trajectory dimension. When the random motion of the particle is slowed-down by the surrounding environment, it results in anomalous subdiffusion, described by 〈**r**^2^(*τ*)〉=*D*_*α*_*τ*^*α*^, where *α*<1 (Fig. 1b). Thus, subdiffusion is an indicator of interactions of the genomic regions with constituents of the nucleoplasm. For better clarity, we plot the MSD divided by time, 〈**r**^2^(*τ*)〉/*τ*, as a function of *τ* on a log–log scale^19^. Normal diffusion appears as a horizontal line (zero slope) and anomalous subdiffusion appears as a negative-slope line of *α*−1 (Supplementary Fig. 2, equations (1, 2, 3, 4)). We also adopted an improved characterization method for extracting the anomalous diffusion parameters^22^.

Previous dynamic measurements showed that the motion of telomeres in normal cells is generally constrained^23^,^24^,^25^. The motion of telomeres in human U2OS cells was characterized as anomalous subdiffusion^21^ and identified as fractional Brownian motion (FBM) that originates from visco-elasticity^26^,^27^.

### Diffusion of genomic loci in the nucleus

**Figure 2:**
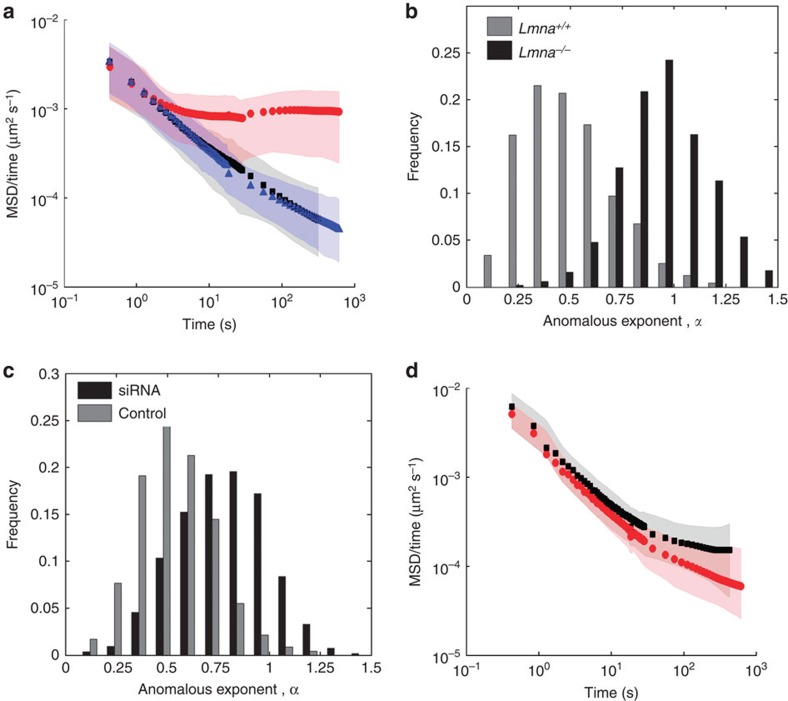
Lamin A depletion changes chromatin diffusion. (**a**) MSDs divided by time for telomeres in *Lmna*^+/+^ cells (black squares, *N*=474), *Lmna*^−/−^ cells (red circles, *N*=503) and *Lmna*^−/−^ cells after transfection with eGFP–pre-lamin A (blue triangles, *N*=220). Symbols designate the average locus MSD while shaded areas mark the s.d. of all single loci MSDs. (**b**) Histograms of the anomalous exponent, *α*, for individual telomeres in *Lmna*^+/+^ (black bars) and *Lmna*^−/−^ cells (grey bars). (**c**) Histograms of *α* values of individual telomeres in U2OS cells after siRNA-mediated knockdown of lamin A (black) and control (grey). A clear increase in the anomalous exponent is found (Student's *t*-test *P*<10^−10^). (**d**) Depletion of LAP2α in MF cells does not change the anomalous diffusion. *Lap2α*^*+/+*^ telomeres in black squares (*N*=551) and *Lap2α*^*−*/−^ telomeres in red circles (*N*=629).

Here we measured the dynamics of various genomic loci—telomeres, centromeres and a gene locus (Fig. 1c, Supplementary Fig. 1) in several cell lines, including U2OS, HeLa, NIH3T3, mouse fibroblasts (MFs) and MEFs (Figs 1d and 2a black squares, Supplementary Fig. 3). In all cases, loci diffusion was found to be anomalous with *α* ranging from 0.4 to 0.7 (Supplementary Table 1). Number of loci replicates for each cell line is given in Supplementary Table 2 and ranged from 166 to 958 for telomeres and centromeres and 20 gene loci. Slow anomalous subdiffusion can thus be regarded as the typical diffusion of genomic sites in the nucleus. This reflects a very slow and localized motion, and it will typically take a chromatin locus several days to diffuse through a chromosome territory that is usually >1 μm across (Supplementary Table 1).

### Diffusion of genomic loci in lamin A-depleted cells

Since lamin A is not only a lamina protein but also a nucleoplasmic protein, whose latter function is unknown, we decided to examine loci diffusion in cells lacking lamin A. Strikingly, in contrast to the slow anomalous diffusion in all *Lmna*^+/+^ cells, the diffusion of telomeres in *Lmna*^–/–^ MEFs was normal for *τ*>7 s with α=1±0.2 where the range signifies s.d. of the population, *σ*_*α*_ (Fig. 2a, red circles, 503 telomeres). This remarkable transition of the diffusion pattern from anomalous to normal is demonstrated in the histograms of individual telomere exponents, *α* (Fig. 2b).

Moreover, expression of transfected enhanced GFP (eGFP)–pre-lamin A in *Lmna*^–/–^ MEFs (Fig. 2a, blue triangles) restored the anomalous nature of telomere diffusion (*α*=0.6±0.1, 220 telomeres). Similar effects were obtained for telomeres and centromeres in U2OS cells in which lamin A was knocked down by short interfering RNA (siRNA) (Fig. 2c and Supplementary Figs 4 and 5, data from 380 and 550 loci, respectively).

### Diffusion of genomic loci in *Lap2*α-depleted cells

To emphasize the unique impact of lamin A on genome dynamics, we measured the effect of lamina-associated-polypeptide LAP2α, that is known to localize in the nucleus and bind lamin A and chromatin^13^, by monitoring telomere dynamics in wild-type (WT) *Lap2*α^+/+^ and *Lap2*α^−/−^ MFs (Fig. 2d). In contrast to the results found for lamin A, telomere diffusion was found to be anomalous in both WT cells (*α*=0.75±0.1, 550 telomeres) and in *Lap2*α^−/−^ MFs (*α*=0.6±0.15, 630 telomeres). Thus, the transformation from anomalous to normal diffusion found for lamin A-depleted cells does not occur by depleting other chromatin-binding proteins in the nuclear interior, such as LAP2α.

**Figure 3:**
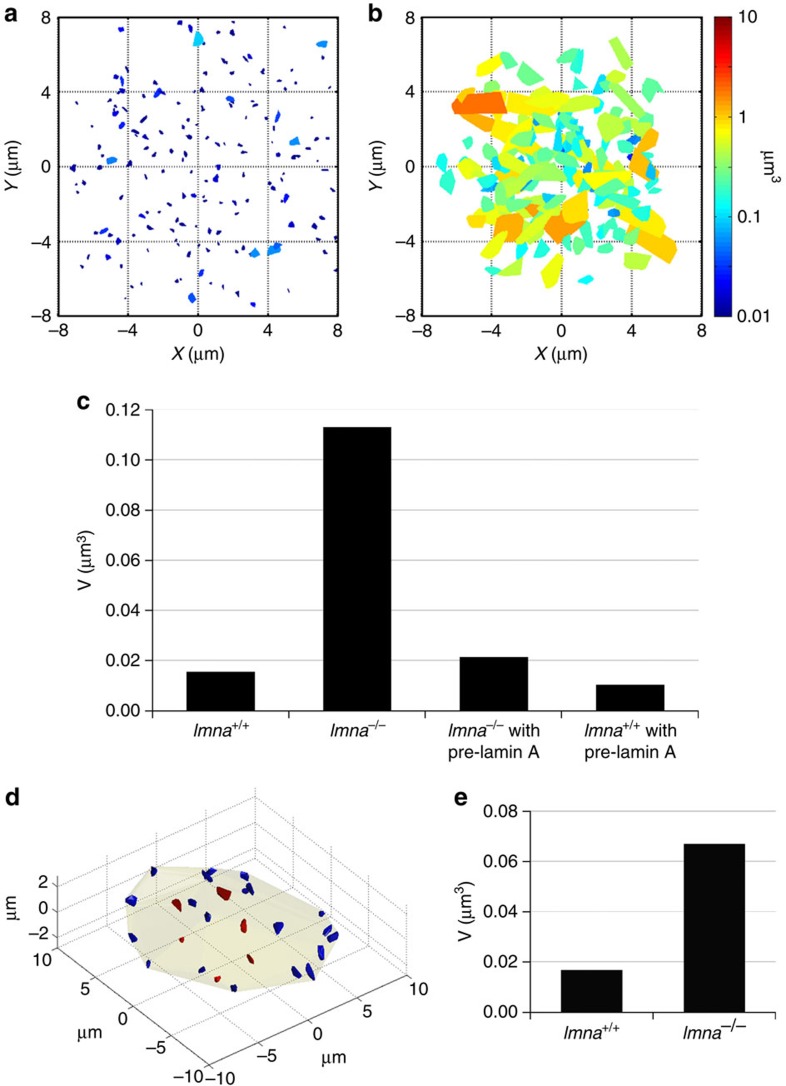
Telomeres scan large areas in lamin A-depleted cells. The area scanned by 350 randomly selected telomeres during 15 min as characterized by the convex hull in (**a**) 15 *Lmna*^+/+^ cells and (**b**) 15 *Lmna*^−/−^ cells. Scanned areas are coloured according to the same logarithmic scale, from 0.01 μm^3^ (blue) to 10 μm^3^ (red). (**c**) Average scanned volumes by telomeres over 15 min in *Lmna*^+/+^ (410 loci), *Lmna*^−/−^ (503 loci) cells and in these same cells transfected with GFP–pre-lamin A (220 loci). Without lamin A, the volume of telomere motion greatly increases. (**d**) Separation between internal (red) and peripheral (blue) telomeres in a U2OS cell. The convex hull of each telomere is plotted as a solid colour volume and the convex hull of all trajectories is presented as a semitransparent volume. Internal telomeres are defined as those that never touch the total convex hull. (**e**) Average volume of movement calculated for internal telomeres in *Lmna*^+/+^ (232 loci in 20 cells) and *Lmna*^−/−^ (135 loci in 20 cells).

Lamin A depletion also led to much faster genome dynamics (Supplementary Movies 2 and 3), as well as larger nuclear areas scanned, as demonstrated by plotting telomere and centromere movement areas in different cells (Fig. 3a–c and Supplementary Table 1).

Altogether, these results reveal an unrecognized role of lamin A in chromatin dynamics. Such an effect on the chromatin dynamics has not been observed so far, and specifically lamin A is the first protein found to induce such an effect.

### Diffusion of genomic loci in ATP depletion conditions

To test if the anomalous subdiffusion results from an active process or through genome packaging, we measured ATP-depleted cells and cells under osmotic stress, (Supplementary Fig. 6, 201 and 214 telomeres, respectively). Although under these conditions telomere diffusion became slower, the diffusion type remained anomalous. Similarly, telomere diffusion in *Lmna*^–/–^ cells under ATP depletion conditions (Supplementary Fig. 6) was slower, but the diffusion type remained normal (231 telomeres). Hence, the properties of chromosome diffusion are primarily not driven by ATP or chromatin condensation.

### Diffusion of internal and peripheral telomeres

One of the proposed mechanisms for genome organization relies on the binding of chromosomes to the peripheral lamina. If that is the dominant mechanism, the dynamic properties of genomic sites should mainly depend on their distance from the lamina. To test this concept, we measured the diffusion of peripheral and interior telomeres (Fig. 3d). In *Lmna*^+/+^ cells both peripheral and interior telomeres exhibited slow and anomalous diffusion, and in *Lmna*^−/−^ cells both showed significantly faster and normal diffusion. Peripheral telomeres showed an average volume of motion of 0.015 μm^3^ in *Lmna*^+/+^ compared with 0.13 μm^3^ in *Lmna*^−/−^ cells (Supplementary Fig. 7), while internal telomeres showed an increase of average volume from 0.017 μm^3^ in lamin-expressing cells to 0.07 μm^3^ in lamin-depleted cells (Fig. 3e). Although binding to the lamina affects the dynamics, it is unlikely that this binding alone can affect the diffusion properties at short times in the nucleus interior. Therefore, it is likely that there is another local mechanism that governs genome dynamics at every region in the nuclear interior.

### Continuous photobleaching of GFP–lamin A

Lamin A at the lamina is highly immobile, which is important for the anchorage of chromatin to the nuclear periphery. To test whether the nucleoplasmic pool of lamin A can affect genome organization not only through anchorage to the lamina, but also locally in the volume of the nucleus, we used continuous photobleaching^28^. The continuous photobleaching technique is based on measuring the fluorescence intensity of labelled proteins (GFP–lamin A) in a specific small region of the live cell. We repeated the measurements in a series of points randomly selected in the nuclear interior. The interplay between the bleaching rate and diffusion that replenishes the bleached fraction enables the characterization of the molecule's dynamics^29^. By measuring the cntinuous photobleaching of eGFP–pre-lamin A in a small confocal spot in the nucleoplasm, we could extract the ratio of free-to-bound lamin A. eGFP–pre-lamin A has been used numerous times and described to have similar dynamic properties during the cell cycle as endogenous lamin A^18^.

**Figure 4:**
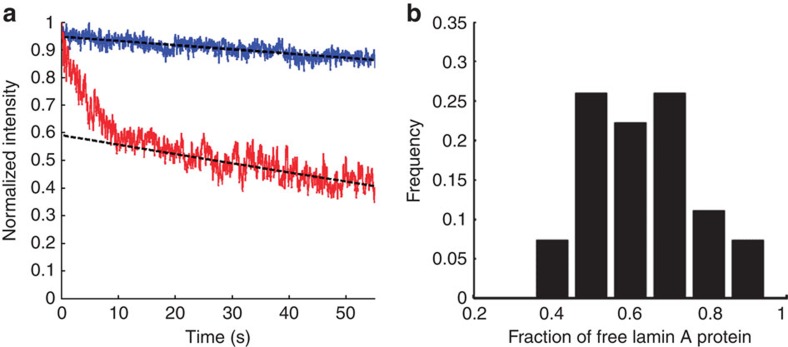
Continuous photobleaching results (**a**) Normalized CP curves of free eGFP protein (blue) and GFP–pre-lamin A in MF cells (red). The intersection of the linear long-time fit (dashed black) and *y*-axis provides the free fraction (59% in the presented curve). (**b**) Histogram of the values of free fraction of eGFP–pre-lamin A in MF cells (27 repeats). The average free fraction is 63±12%.

The fraction of freely diffusing lamin A in the nucleoplasm was found to be 63±12% (Fig. 4, 27 cells), which means that a significant fraction (∼40%) of bound immobile lamin A is found in the centre of the nucleus. This stable fraction of lamin A can therefore govern anomalous chromatin diffusion by direct or indirect interaction with chromatin and provides strong evidence to the local mechanism by which lamin A governs chromatin dynamics.

### Diffusion of telomeres in cells expressing mutated lamin A

Finally, to further characterize the effect of lamin A on chromosome dynamics, we measured the influence of mutated lamin A proteins linked to different diseases^30^ (Supplementary Fig. 8 and Supplementary Table 3). Four of these disease mutants were expressed in *Lmna*^–/–^ cells, each with a different mutation in the rod (L85R and N195K) or tail (R482W and L530) domains of the lamin A protein. For each mutant, we measured and analysed over 500 separate trajectories of telomeres. In all four cases the telomere diffusion characteristics were corrected only partially towards the diffusion characteristics in *Lmna*^+/+^ cells. This is in contrast to expression of WT eGFP–pre-lamin A in *Lmna*^–/–^ cells which fully retrieved the anomalous diffusion in *Lmna*^+/+^ (Fig. 2a). We conclude that both rod and tail domains of lamin A are responsible for maintaining proper chromosomal organization. Since the rod domain of lamin A is responsible for formation of the protein dimer, and the tail domain for interaction with histones and DNA^31^, this suggests a possible crosslinking mechanism of two chromatin strands through lamin A protein oligomers.

Lamin A depletion leads to a transition from slow anomalous diffusion to fast normal diffusion for *τ*>7 s and distances beyond ∼100 nm. Polymer diffusion models predict anomalous diffusion of monomers up to the length scales of the polymer globule (here a chromosome territory of size *r*∼1 μm)^5^,^32^. More than this, fast normal diffusion is not expected for polymers in dense visco-elastic environments. This strongly suggests that a polymer diffusion model by itself is not sufficient for explaining the results and another mechanism is needed. Polymer models, however, can explain the short time-range anomalous diffusion that is measured with or without lamin A^33^.

## Discussion

**Figure 5:**
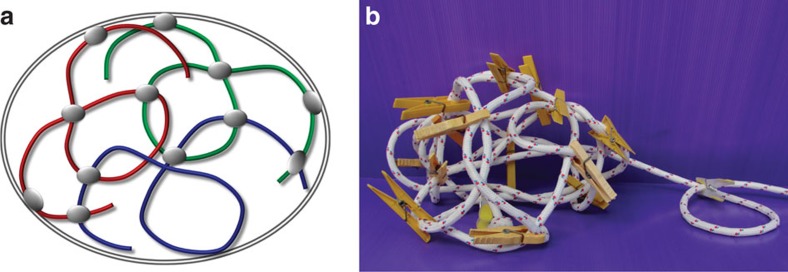
Model for DNA crosslinking. (**a**) Schematic model suggested for the nucleus organization. Chromosome cross-links formed in and between chromosomes by lamin A or a complex that contains lamin A. Anchoring to the lamina is also shown. (**b**) Toy model of the suggested mechanism, showing how a rigid structure arises from a cross-linked flexible polymer (that is, rope).

We have shown that lamin A has a unique function in regulating chromatin dynamics, with crucial roles of both head and tail domains. Lamin A is present in the nucleoplasm and not only at the lamina^18^, in agreement with our results showing that a significant fraction of lamin A is bound throughout the whole nucleoplasmic volume. Accordingly, we propose that genome organization is maintained by cross-linker-binding events through lamin A (or complexes that contain lamin A) in the nuclear interior, and not only through chromosomal anchoring to the peripheral lamina (Fig. 5).

In this model, chromatin itself acts as the major building block that turns into a constrained polymeric structure when cross-links are formed. Such a structure would preserve the anomalous diffusion at large time and length scales, as theoretically suggested before^10^,^34^ and naturally explains the maintenance of chromosome territories. This model is supported by the fact that lamin A accumulates in the nucleoplasm and not only at the nuclear envelope^18^ and forms oligomers that can cross-link chromatin either by direct binding to the DNA, or through the H2A/H2B core histone proteins^15^,^35^. The model also complies with our results on mutated lamin A proteins and CP data. Finally, our model is consistent with recent studies reporting that chromatin movement was coherent across large regions (4–5 μm) for several seconds^36^, regions that extend beyond the boundaries of a single chromosome, and suggested an elastic coupling in these large regions. In accordance with our model, visco-elastic behaviour was measured and should be an outcome of the chromosomal crosslinking that we suggest.

The effect of lamin A on chromatin organization may be direct, as suggested above, or, alternatively, it is also possible that lamin A depletion influences chromatin dynamics through the modification of expression levels of various other structural proteins. This should be addressed in future studies. In addition, it would be interesting to compare chromatin dynamics in proliferating and differentiated cells, where the expression profiles of lamin A are different. Finally, our model also enables cells to control chromatin dynamics locally according to transcriptional and cell cycle demands, by local unbinding of lamin A.

## Methods

### Cell culture

Human U2OS osterosarcoma cells were maintained in Dulbecco's low glucose-modified Eagle's medium. NIH3T3 and HeLa cells were maintained in Dulbecco's modified Eagle's medium (Biological Industries, Israel). Medium contained 10% of bovine serum, 1% of penicillin and streptomycin antibiotics (Biological Industries). For studying the dynamics of a gene locus, we used a U2OS Tet-On cell line with stable transfection of lacO repeats that enable the fluorescent marking with red fluorescent protein (RFP)–LacI of the genomic locus of integration of a human β-globin gene^37^. Cells used in this study also include knock out *Lap2α*^−/−^ MFs and their WT *Lap2α*^+/+^ MFs^38^, MEFs lacking lamin A/C (*Lmna*^−/−^ MEFs) and their WT (*Lmna*^+/+^) MEFs^39^. ATP depletion was carried out by incubation of cells for 30 min in 50 mM deoxy-glucose (Sigma-Aldrich) and 10 mM of sodium azide (Sigma-Aldrich) prior to the imaging^40^. For the osmotic treatment, 100 μl of 10 × PBS was added into 900 μl of culture medium^41^. Imaging was performed 5 min after PBS addition and lasted no more than 1 h.

To check the restoration of telomeres motion by expression of laminA, cells that do not express lamin A (*Lmna*^*−/−*^) were transfected with two plasmids eGFP–pre-lamin A (for lamin A restoration) and DsRed-TRF1 (telomere labelling). Cells that expressed both proteins were imaged and telomere's motion was analysed. The impact of mutant lamin A variants was examined in the same method of dual plasmid transfection.

### Plasmids

GFP–TRF2 plasmid was a kind gift from Sabine Mai (Manitoba Institute of Cell Biology, Canada); CENPA–eGFP and TRF1-DsRed were kind gifts from Vered Raz (Leiden University Medical Center, The Netherlands). LacI–RFP was provided by Yaron Shav Tal; eGFP–pre-lamin A was provided by Suzana Gonsalo (Saint Louis University School of Medicine, USA). pcDNA3–GFPLaminA-L85R (Addgene plasmid 32707), pcDNA3-GFPLaminA-N195K (Addgene plasmid 32708), pcDNA3-GFPLaminA-R482W (Addgene plasmid 32709) and pcDNA3-GFPLaminA-L530P plasmids (Addgene plasmid 32710) were generated in the lab of Wendy Bickmore^42^.

### Immunofluorescence

Cells were grown on glass coverslips and fixed with 4% paraformaldehyde (20 min) followed by extraction in 0.5% Triton X-100 in PBS for 3 min at room temperature. Slides were then washed twice in PBS and blocked in 5% BSA for 20 min. Antibodies: lamin A/C (Abcam, ab26300, dilution 1:400). Secondary antibodies: anti-rabbit Alexa-488 (Molecular Probes, A11034, dilution 1:1,000). DNA was stained with Hoechst 33258 (Sigma-Aldrich). Slides were imaged on an inverted Olympus IX-81 fluorescence microscope.

### siRNA silencing

siRNA sequences were produced by IDT technologies. The following target sequences were used: Lamin A/C: 5′-AGCUGAAAGCGCGCAAUACCAAGAA-3′; Control siRNA was performed with negative control sequences (IDT). RNA-mediated interference was performed on U2OS cells at 75% confluence grown on glass bottom dishes. For each dish, 10 nM siRNA in 200 μl Optimum medium without serum (Gibco) was mixed with 2 μl of Lipofectamine 2000 (Life Technologies). Cells were imaged 72 h after transfection with the siRNA. Total RNA was extracted with Aurum Total RNA Mini Kit (Bio-Rad Laboratories). Complementary DNA (cDNA) was prepared with qScript cDNA SuperMix (Quanta BioSciences). Primers used for lamin A reverse transcription–PCR: 5′-AGC AGC GTG AGT TTG AGA GC-3′ and 5′-AGA CTG CCT GGC ATT GTC C-3′. Gene expression level was normalized to GAPDH and Actin.

### Imaging

Cells were placed in a 37 °C incubator (Tokai) with a 5% CO_2_ level on an inverted Olympus IX-81 fluorescence microscope coupled to a FV-1000 confocal set-up (Olympus) using a UPLSAPO × 60 objective, numerical aperture=1.35. Short times (0.5–100 s) were measured in 2D by focusing on a single plane in the nucleus which exhibited multiple fluorescent loci. At time longer than 100 s, stage drift in the optical axis becomes significant and demands measuring in 3D to correct the trajectories (see ‘image analysis'). Volumetric whole nucleus measurements were performed on a Freerun imaging set-up, giving an image every 18.5 s. The whole nuclear volume was covered in addition to focal planes above and below the nucleus to capture stage drift. Most experiments were measured for 100 time points (that is, up to 31 min) yet some experiments showed increased bleaching and were measured for 50 time points (*Lmna*^+/+^ MEFs, *Lmna*^–/–^ MEFs with ATP depletion and *Lap2α*^*−*/−^ MFs). For these shorter experiments, we measured more cells to compensate for reduced temporal statistics.

The measurement precision was estimated to be ∼20 nm by measuring fixed U2OS cells labelled for telomeres, using the same imaging technique as above. The total number of measured loci for each cell line and treatment is described in Supplementary Table 2.

### Image analysis

Quantitative SPT analysis of time-lapse image sequences was performed using the Imaris (Bitplane) image analysis software package for locating coordinates of labelled genomic loci. For our analysis, we used only trajectories that had no missing time points and checked each trajectory to validate that there was no concatenation between different loci.

Nucleus drift and rotation correction were performed in two stages. First, the centre of mass location for all loci in each frame is subtracted from the trajectories. This corrects for drift. Then, the average rotation matrix in the imaging plane for all loci around the centre of mass is calculated for each time point. Finally, each trajectory is multiplied by the inverse rotation matrix. Our cells are all surface adhering and no out-of-plane rotation (that is, around an axis in the imaging plane) is seen.

To correct the motion of the nucleus for the gene locus, GFP–TRF2 and RFP–LacI were co-expressed in U2OS cells. The gene location was corrected according to the nucleus drift and rotation results were obtained from the co-observed telomeres.

### Data analysis

#### Diffusion analysis

The time-averaged MSD was calculated for each genome locus by finding the average squared displacement between each two time points with a time interval *τ=n. δt,* where *δt* is the measurement time interval and *n* is an integer. Averaging is performed over the entire measured time,


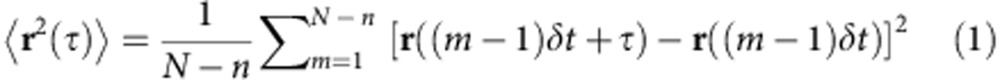


Here, 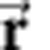 is the 2D position vector of the particle at each time point and *N* is the total number of time points measured. When analysing the MSD, we used only the *x–y* plain motion as the *z*-axis (confocal axis) has higher measurement errors and is assumed independent.

Normal diffusion shows an MSD that grows linearly with time, 〈**r**^2^(*τ*)〉=2*dDτ*, where *d* is the dimension in which the motion is performed and *D* is the diffusion coefficient. For anomalous diffusion, this fundamental characteristic is violated. When the motion is according to a temporal power law, 〈**r**^2^(*τ*)〉=*D*_*α*_*τ*^*α*^ then another classification is used. If the anomalous exponent *α*<1 then the motion is anomalous sub-diffusive, and if *α*>1 the motion is super-diffusive. Notice that the generalized diffusion coefficient has units that are dependent on the anomalous exponent, specifically in our case—μm^2^ s^−*α*^. Thus, it is impossible to compare the diffusion coefficient between processes that have different exponents.

To make the qualitative analysis of MSD graphs easier, it is common to present them on a log–log scale. When this is done, the slope of the presented graph is the anomalous coefficient *α*. If an easy differentiation between normal and anomalous diffusion is requested, it is customary to divide the MSD by *τ*. In these plots, the slope is *α*−1 (Equation 2, 3, 4). Normal diffusion translates to a zero slope, anomalous super-diffusion is positive and anomalous subdiffusion is negative. The height of the curve in the log–log graph is the diffusion constant (*D* in normal diffusion), thus also allowing easy differentiation between diffusion constants (Supplementary Fig. 2).


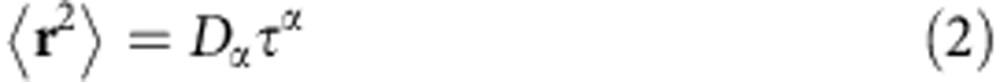



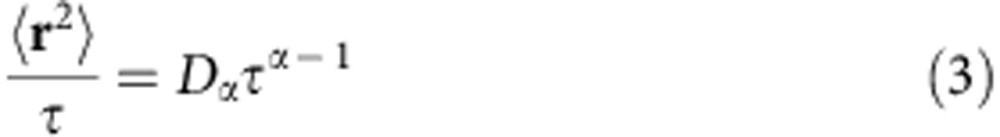



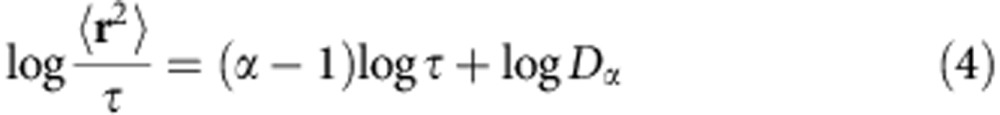


Accurate extraction of the anomalous exponent is a crucial step in characterizing anomalous processes. However, the heterogeneous nature of biological processes and the errors in localization of fluorescent entities introduce time-dependent errors into the results. In fact, it is impossible to extract the value of *α* for short trajectories (measurement points <10^3^) without significant errors even by averaging the individual MSDs. Hence, single particle MSDs were used to compare between populations and not extract absolute values.

A previously described method^22^ corrects for these errors and extracts both the average and the variance of the distribution of *α* for the ensemble of particles. A detailed description of this technique is given in the next section.

### Quantification of population diffusion characteristics

A series of previous studies^26^,^27^, have shown that telomere motion in U2OS cells obeys, to first order analysis, fractional Brownian motion (FBM). FBM is a generalization of normal Brownian motion that can account for a non-Markov nature of a diffusion process, that is, correlations in time. For loci moving according to FBM, a previously published technique^22^ can be implemented for the analysis of the MSDs and to overcome the inherent problems of simple power law fitting. The mean logarithmic square displacement (MLSD) technique has been shown to correct for localization errors and the tendency of trajectories with large anomalous exponents to dominate the ensemble of particle MSDs. Since it is not possible to accurately analyze individual trajectories that are shorter than 10^4^ time points, it was necessary to extract the diffusion parameters through the simultaneous analysis of hundreds of trajectories.

The general line of the methodology used is:

1. Compute the time-averaged MSD for every trajectory, 〈**r**_*i*_^2^〉.

2. Measure the s.d. of the measurement error on static loci *ρ*.

3. Extract the anomalous exponent of the mean time-averaged MSD and the MLSD:


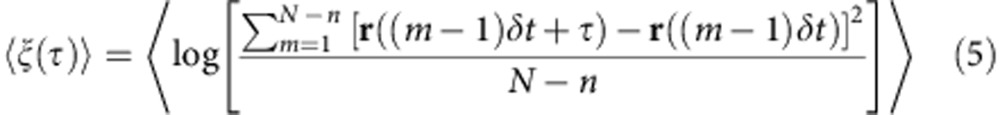


4. Compare the anomalous exponents and fit the differences *ɛ*=*α*_MSD_−*α*_MLSD_ to 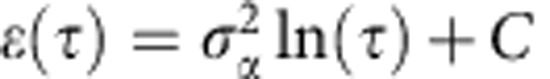. This gives the variance of the anomalous exponent 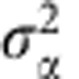.

5. Find the time average MSD of the average particle with:


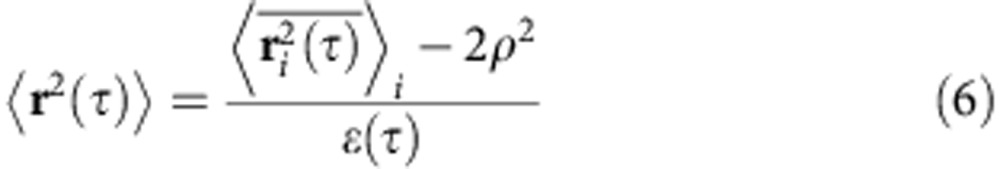


and fit 〈**r**^2^(*τ*)〉 to find the average 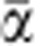 and *D*_*α*_.

The results stated in the manuscript and in the Supplementary Information designate the parameters extracted for the ensemble of the loci analysed, and rounded to nearest tenth decimal digit.

### Separation between interior and peripheral loci

Separation between peripheral and interior loci was performed based on the convex hull created by the diffusion of loci in the nucleus. For each nucleus, at each time point, the convex hull was calculated as the set of planes, of minimal area, that confine all loci. The loci that do not touch the convex hull are defined as the internal loci for that time point, {*I*_*t*_}, and the ones that do are defined as peripheral loci for that time point, {*P*_*t*_} (Fig. 3d). After analysing each time point *t*_*i*_, we define the final internal loci as those who never touched the convex hull 
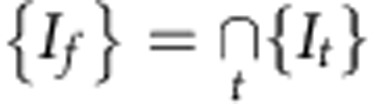
, while the rest are termed the final peripheral loci for that cell, 
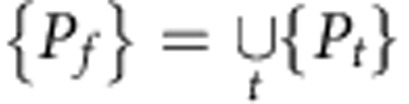
. These two separate groups can then be analysed separately, while in all likelihood the internal loci are those that have never been close to the nuclear lamina during our experiment.

### Continuous photobleaching

CP is performed by using a confocal microscope set-up. After measuring the image, a specific point is selected, and its intensity is measured at a high frequency (∼1 KHz) for a given amount of time. Due to bleaching, the measured intensity reduces, but the actual time-dependent function depends on the nature of measured protein. Assuming it has two different populations that are being measured simultaneously: a bound fraction and a free fraction. The free fraction of the molecules diffuses through the measured point and their intensity only slowly bleaches, see, for example, the CP curve of free GFP (Fig. 4a, blue curve). In contrast, the bound fraction stays in the measured spot and bleaches much faster. Therefore, if both populations exist, the CP curve starts with an exponential decay (of the bound fraction) and continuous with a slowly decreasing linear curve, see, for example, the lamin A CP curve (Fig. 5a, red curve). By analysing the curve one can extract the ratio of the bound and free fraction of the protein. If the binding−unbinding time-scale of the bound proteins is large relatively to the diffusion time of the free proteins through the measured spot, the analysis is straightforward (see below).

The measurement was performed on a confocal microscope (Olympus, FV-1000) on an inverted microscope, the same one that was used for all the other measurements reported here. It is important to use sensitive detectors and we therefore used a Picoquant Microtime 200 (MT200) set-up combined with the FV-1000 confocal microscope. The MT200 system uses a 20-MHz 470-nm pulsed picosecond diode laser (LDH-P-C-470B, PicoQuant GmbH, Berlin, Germany). The light is coupled to the FV-1000 via an optical fibre and focused onto a small confocal volume through a × 60 water immersion objective lens with numerical aperture=1.2 (UPIanSApo, Olympus). The emitted light was collected through the objective, filtered from the excitation light through an appropriate dichroic mirror (405/488 nm), transmitted through a confocal pinhole (*D*=120 μm) and sent to a single photon avalanche detector (SPAD, Perkin Elmer SPCM-AQRH 13) through a 520/35-nm Band-pass filter (FF01-520/35-25, Semrock, Rochester, NY, USA).

The laser intensity was set to ∼2.7 μW at the back aperture of the objective and a measurement of the cell was performed with the confocal microscope. Then, a specific point was chosen in the nuclear interior and a ‘point measurement' was performed for ∼60 s with the MT200 SPAD detector. Finally, the measurement is repeated in other cells.

CP data is a fluorescent intensity measured at a certain point as a function of time. The excitation laser slowly bleaches the FPs at the active optical spot while simultaneously exciting them. Therefore, the fluorescent signal that is detected is proportional to the un-bleached FP's that are still in the optical volume. CP data analysis was performed with the Matlab programme and an algorithm we wrote.

For analysing the data, we assume that the time-evolution of the labelled molecule concentration includes an immobilized bound fraction of molecules *C*_immo_(*r*,*t*) with a *k*_on_ binding and *k*_off_ unbinding time, as well as a freely diffusing fraction *C*_diff_(*r*,*t*) with a diffusion coefficient *D*. These concentrations change with time according to the following conjugated equations^28^:


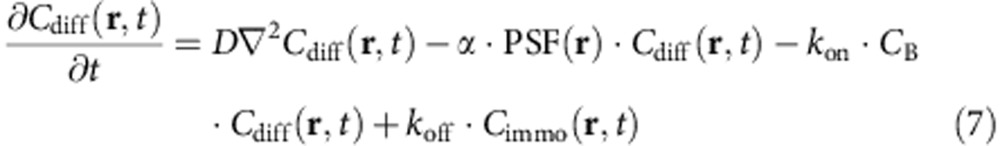



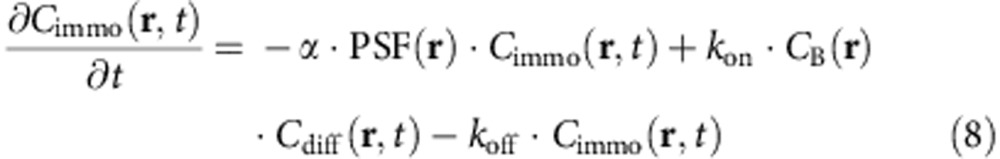


where *α* is the bleach rate at the centre of the illumination spot, *C*_B_ is the concentration of binding sites and PSF(*r*) is the intensity distribution of the point spread function (PSF) of the confocal laser. These equations can be solved numerically and the relevant constants can be extracted. Moreover, it can be shown that if the binding-unbinding time is slower than the typical time for the molecule to diffuse through the confocal spot, the fluorescence intensity can be fitted to a sum of an exponential decaying term that corresponds to the bleaching of the bound fraction and a linear slowly decaying term that corresponds to the diffusing fraction.

Accordingly, the measured data was first smoothed within a 0.1-s window. Then the experiment starting point (*t*=0, when the laser was turned on) was determined based on the maximal derivate of the intensity trace. The intensity trace was fitted to the model equation *I*(*t*)=*ae*^−*bt*^+*ct*+*d* and the ratio free/total was calculated as the ratio between the extrapolated value of the intensity linear term (*d*, the intensity of the free fraction at *t=0*) and the intensity value at the beginning of the measurement, *t*=0, *I*(0)=*a*+*d*. A rather good fit is found in all the different measurements. Note also that the parameter *α* that determines the bleaching rate can be matched to the conditions so that the simplified model is adequate. Moreover, if the binding/unbinding times are in the same range as the diffusion time across the illuminated spot, it can be shown that the extracted immobile fraction is the lower limit of the actual immobile fraction. One can recognize that if these times are similar, the bound fraction never bleaches completely, and contributes to the total fluorescence even at longer times, which leads to the described result. Numerical solution of the equations also supports this result.

## Additional information

**How to cite this article:** Bronshtein, I. *et al.* Loss of lamin A function increases chromatin dynamics in the nuclear interior. *Nat. Commun.* 6:8044 doi: 10.1038/ncomms9044 (2015).

## Supplementary Material

Supplementary Information Supplementary Figures 1-8, Supplementary Tables 1-3 and Supplementary Reference
