# Supplementary material for: Loss of lamin A function increases chromatin dynamics in the nuclear interior
Source: Nat Commun. 2015 Aug 24;6:8044. doi: 10.1038/ncomms9044 (PMC4560783; doi:10.1038/ncomms9044)
Supplement: Supplementary Information — Supplementary Figures 1-8, Supplementary Tables 1-3 and Supplementary Reference [file ncomms9044-s1.pdf]

<u>Supplementary Information</u>

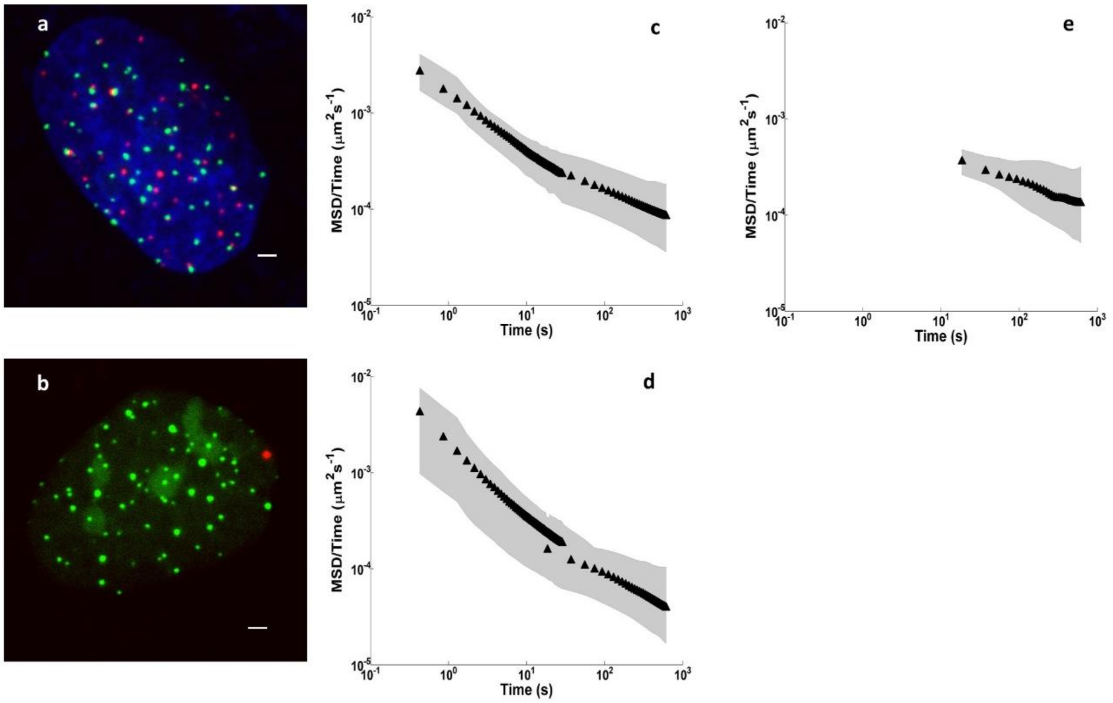

Supplementary Figure 1: Quantitative diffusion analysis of different genomic loci. Visualization of telomeres, centromeres and a gene locus: (a) Projection image of 3D data of labeled centromeres and telomeres by expression of CENPA-eGFP and TRF1-DsRed, respectively and Hoechst staining in blue. (b) Projection image of 3D data of a labeled gene locus and telomeres by expression of RFP-LacI (red) and GFP-TRF2 (green), respectively. Scale bar is $1\mu m$. MSD/Time vs Time in Log-Log scale for: telomeres (c), centromeres (d), and a gene locus (e) in the U2OS cell line. Average loci data is shown as black triangles. Shaded areas mark the standard deviation of single loci MSDs. Trajectories derived from 20-50 cells. Extracted values of α are provided in Supplementary Table 1 and number of measured loci in Supplementary Table 2.

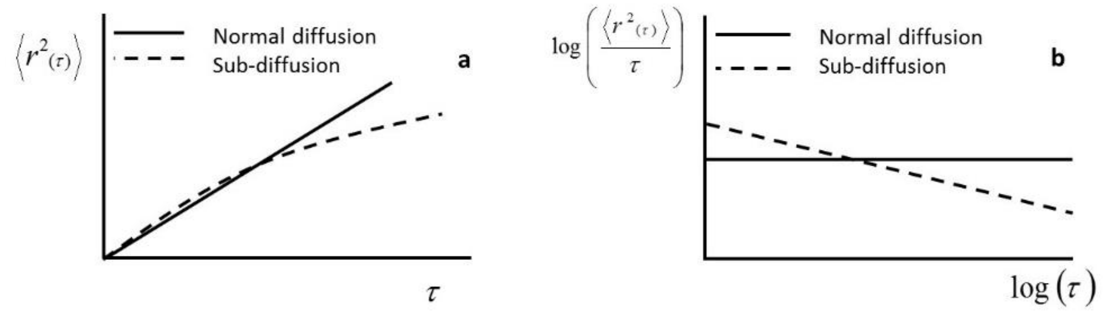

Supplementary Figure 2: Anomalous and normal diffusion with logarithmic plotting. (a) On a normal axis, it is not immediate to differentiate between normal diffusion and subdiffusion. On a logarithmic plot, both are transformed into linear curves with different slopes. (b) Further division of the MSD by the time turns normal diffusion into a constant line and subdiffusion into a negative sloped linear curve.

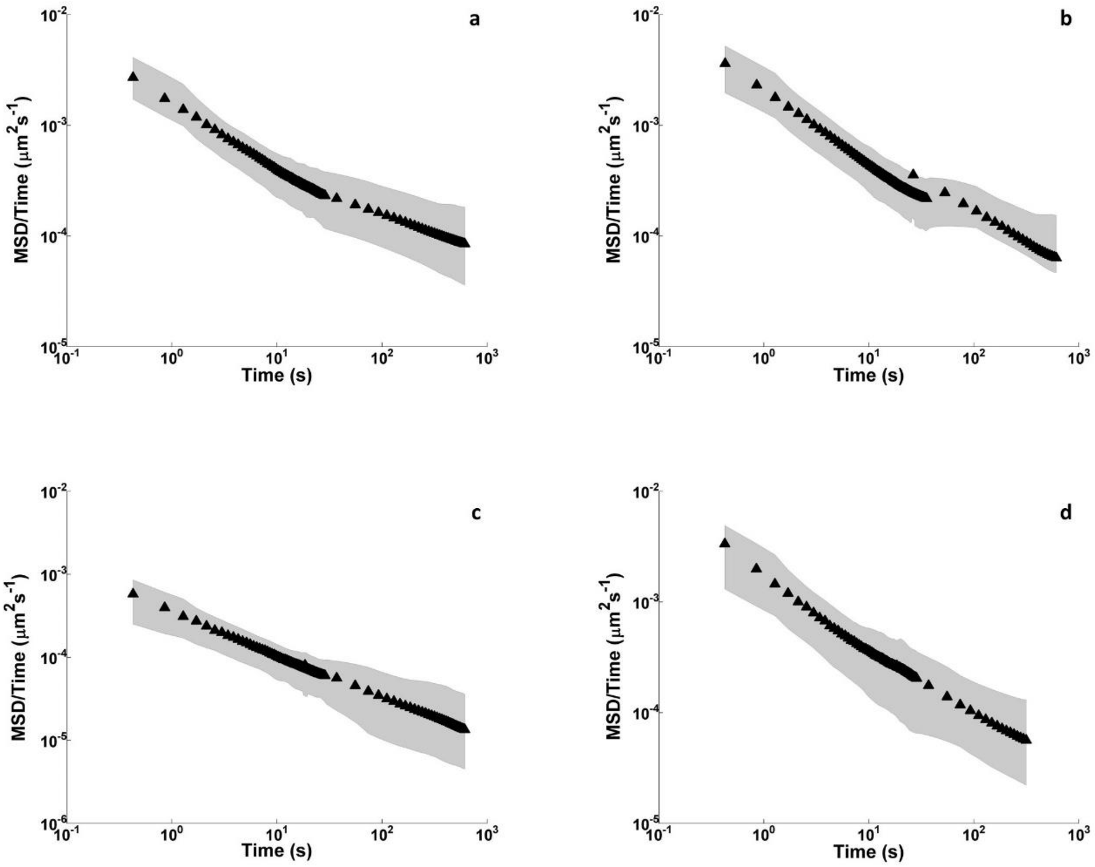

Supplementary Figure 3: Quantitative analysis of telomere diffusion in different cell lines.

MSDs of telomere motion in (a) U2OS cells, (b) HeLa cells, (c) NIH3T3 cells, and (d) MEFs. Average loci data is shown as black triangles. Shaded areas mark the standard deviation of single loci MSDs. The data were measured and analyzed in two time ranges (2D-confocal, 3D confocal) for 100-300 telomeres for each cell line at each time range. Note that y-axis values of NIH3T3 cells is lower than the axes in the other graphs by a factor of ten. Extracted values of α are provided in Supplementary Table 1 and number of measured loci in Supplementary Table 2.

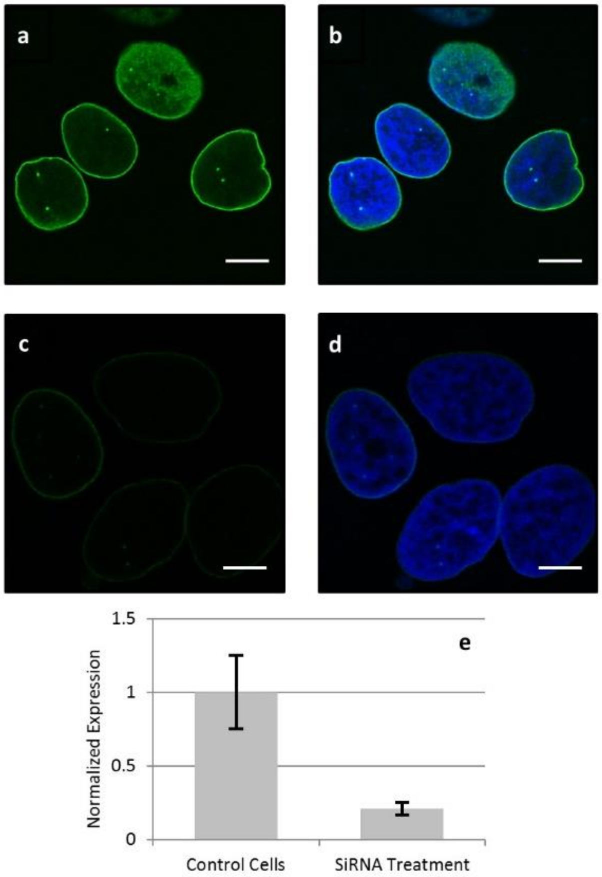

Supplementary Figure 4: Lamin A expression in U2OS cells after treatment with siRNA against lamin A. Upper panels (a-b) - control cells. Lower panels (c-d) - image of cells transfected with siRNA against lamin A. Green – antibody staining for lamin A. Blue – nuclear staining with Hoechst 33258. Scale bar, 10 µm. (e) Expression of lamin A after incubation with siRNA against lamin A measured by RT-PCR. Error bars are standard error of the mean from 3 independent experiments.

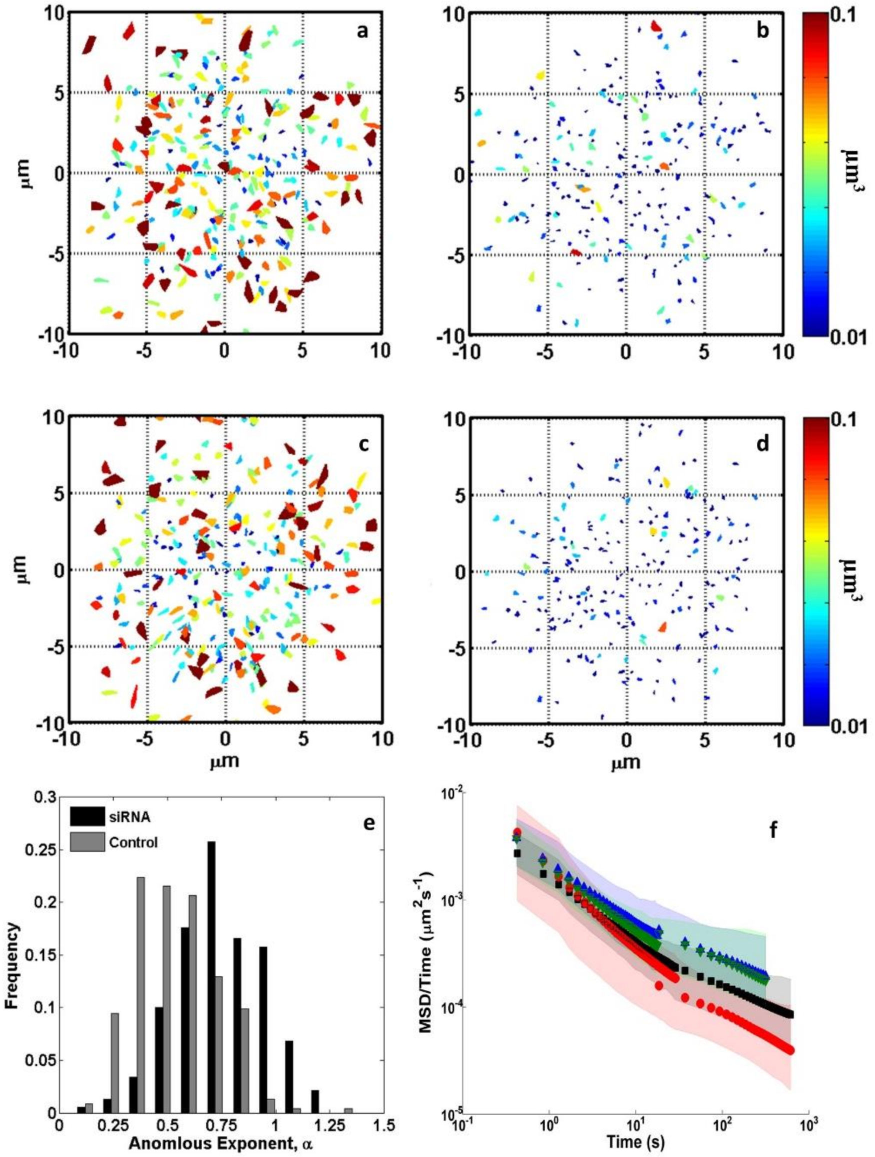

Supplementary Figure 5: Diffusion characteristics of telomeres and centromeres in lamin A-depleted U20S cells. (a) Movement volume during 925 seconds for telomeres in cells transfected with siRNA against lamin A and (b) cells transfected with the negative control. The same behavior appears in centromeres with (c) siRNA against lamin A and (d) a negative control. Each panel shows results from 350 random loci. Note that the color bar is logarithmic. (e) Histograms of α values calculated for individual telomeres (see main text for centromere histogram). Data represents values derived from 15-20 cells, 350-550 loci. siRNA and control distributions are statistically different with student's t-test giving $p < 10^{-10}$. (f) Comparison of MSD divided by time for telomeres and centromeres under depletion of lamin A with siRNA (blue triangles and green inverted triangles respectively) versus telomeres and centromeres in normal conditions (black squares and red circles respectively). Upon lamin A depletion, an increase in diffusion rates and a transition towards normality is seen. See supplementary Tables 1-2 for α values and repeat numbers.

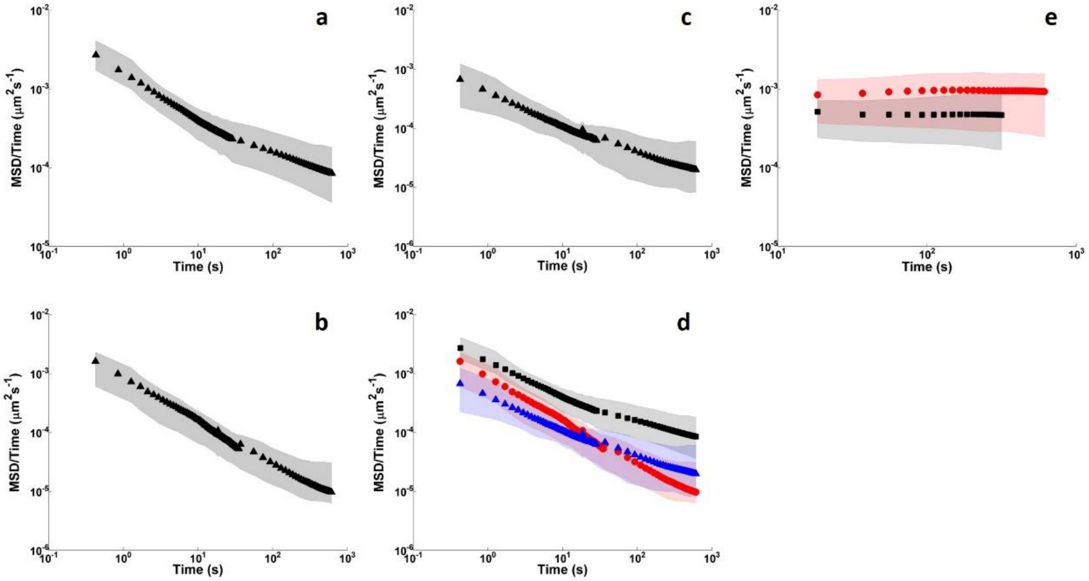

Supplementary Figure 6: Telomere diffusion in the presence of ATP depletion and osmotic stress.

MSDs of telomere motion in U2OS cells under (a) normal conditions, (b) ATP depletion, and (c) osmotic pressure. Average loci data is shown as black triangles. Shaded areas mark the standard deviation of single loci MSDs. While all MSDs are sub-diffusive at all time-spans (d), the average MSD decreases under ATP depletion (red circles) and osmotic pressure (blue triangles) compared to the normal behavior (black squares), (e) MSD of telomeres in Lmna$^{-/-}$ cells under regular conditions (red circles) and after ATP depletion (black squares). Extracted values of α are provided in Supplementary Table 1 and number of measured loci in Supplementary Table 2.

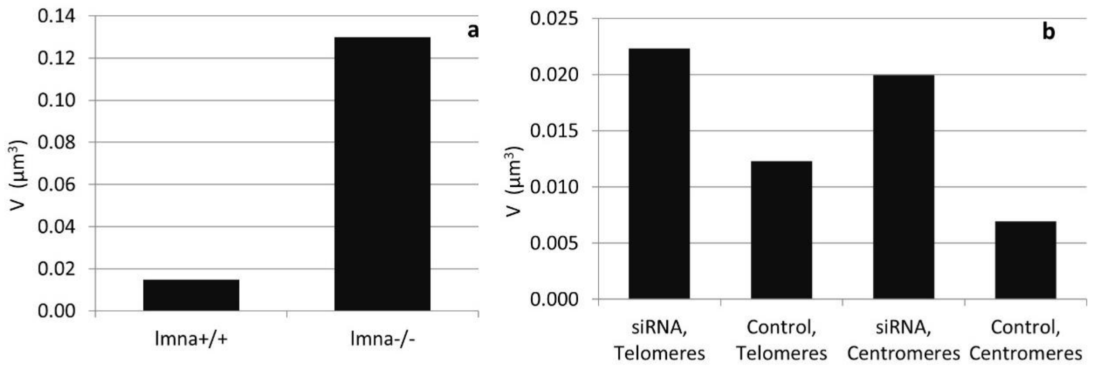

Supplementary Figure 7: Effect of lamin A depletion on telomere's volume of movement.

(b) Average volume of movement calculated for peripheral telomeres in $Lmna^{+/+}$ (410 loci in 20 cells) and $Lmna^{-/-}$ cells (369 loci in 20 cells). (c) Average volume of movement for telomeres and centromeres (21 cells each) under siRNA treatment against lamin A in U2OS cells. A significant difference (student's t-test $p < 10^{-10}$) from controls is seen (10 cells each).

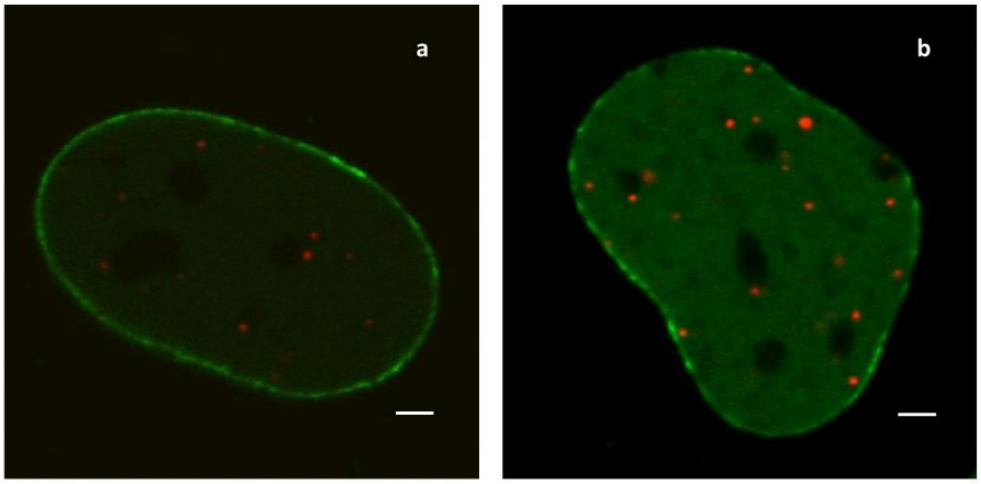

Supplementary Figure 8: Mutant lamin A protein expression in Lmna$^{-/-}$ cells.
Expression of GFP-Lamin A-L85R (a) and GFP-Lamin A-N195K (b) in Lmna$^{-/-}$ cells. Expression of pcDNA3-GFP-Lamin A-R482W and pcDNA3-GFP-Lamin A-L530P is similar to GFP-Lamin A-L85R (6). Telomeres labeled with DsRed-TRF1. The results are summarized in supplementary Table 3. Scale bar, 2 μm

Supplementary Table 1: Summary of anomalous exponents and diffusion coefficients.

Cells	Locus	Treatment	Approximate Time to Diffuse 1 μm^2 [a,b]	10^1-10^3 seconds			10^{-1}-10^1 seconds		
				Average α	σ_α [c]	D_α $[\mu m^2 s^{-\alpha}]$x10^{-4} [d]	Average α	σ_α [c]	D_α $[\mu m^2 s^{-\alpha}]$x10^{-4} [d]
U2OS	Telomere		30 h	0.55	0.16	17	0.38	0.10	16
U2OS	Telomere	ATP depletion	~1011 y	0.25	0.20	9	0.23	0.05	9
U2OS	Telomere	Osmotic stress	186 d	0.47	0.20	4	0.4	0.10	4
U2OS	Centromere		11 d	0.5	0.15	10	0.19	0.10	21
U2OS	Gene locus		4 h	0.75	0.10	8			
U2OS	Telomere	siRNA against lamin A	7 h	0.65	0.2	14	0.43	0.15	22
U2OS	Telomere	siRNA control	22 d	0.47	0.15	11			
U2OS	Centromere	siRNA against lamin A	3 h	0.75	0.15	10	0.4	0.10	21
U2OS	Centromere	siRNA control	43 d	0.45	0.20	11			
MEF $Lmna^{+/+}$	Telomere		50 d	0.43	0.15	14	0.26	0.10	18
MEF $Lmna^{-/-}$	Telomere		24 m	1	0.20	7	0.52 [e]	NA	18

Cells	Locus	Treatment	Approximate Time to Diffuse 1 μm^2 [a,b]	10^1-10^3 seconds			10^{-1}-10^1 seconds		
				Average α	σ_α [c]	D_α $[\mu m^2 s^{-\alpha}]$x10^{-4} [d]	Average α	σ_α [c]	D_α $[\mu m^2 s^{-\alpha}]$x10^{-4} [d]
MEF $Lmna^{-/-}$	Telomere	ATP depletion	30 m	1	0.10	5			
MEF $Lmna^{-/-}$	Telomere	GFP-pre-lamin A transfection	5 d	0.57	0.10	6	0.3	0.10	18
WT MF ($Lap2\alpha^{+/+}$)	Telomere		6 h	0.76	0.05	5	0.12	0.05	30
MF $Lap2\alpha^{-/-}$	Telomere		40 h	0.62	0.15	7	0.6	0.10	24
NIH3T3	Telomere		245 d	0.47	0.15	4	0.43	0.05	4
NIH3T3	Telomere	ATP depletion	~1000 y	0.19	0.20	2	0.02	0.05	5
HeLa	Telomere		16 d	0.43	0.15	23	0.34	0.10	21

Remarks:

a) Units for diffusion times: m -minutes, h - hours, d - days, y – years.

b) All results are approximate and are extracted through $T = (1/D_\alpha)^{1/\alpha}$.

c) The standard deviation of anomalous exponents for the whole ensemble of measured loci. Extracted using the MLSD technique[1].

d) Diffusion coefficients are for two-dimensional motion. For Brownian motion the following relation exists $D_{1d} = D_\alpha / 4$ since $D_\alpha = 2dD_{1d}$ (where d is the dimension of the random walk). However, this is not the case for general anomalous diffusion.

e) At the shortest times, the diffusion exponent is $\alpha \approx 0.5$. At larger times there is a transition to normal diffusion with $\alpha \approx 1$.

Supplementary Table 2: Number of loci measured in each data set.

Cells	Locus	Treatment	Loci Measured
U2OS	Telomere	NA	958
U2OS	Telomere	ATP depletion	201
U2OS	Telomere	Osmotic stress	214
U2OS	Centromere	NA	957
U2OS	Gene locus	NA	20
U2OS	Telomere	siRNA against lamin A	380
U2OS	Telomere	siRNA control	232
U2OS	Centromere	siRNA against lamin A	551
U2OS	Centromere	siRNA control	235
WT MEF (Lmna$^{+/+}$)	Telomere	NA	474
MEF Lmna$^{-/-}$	Telomere	NA	503
MEF Lmna$^{-/-}$	Telomere	ATP depletion	231
WT MF (Lap2α$^{+/+}$)	Telomere	NA	551
MF Lap2α$^{-/-}$	Telomere	NA	629
NIH3T3	Telomere	NA	325
NIH3T3	Telomere	ATP depletion	187
HeLa	Telomere	NA	166
MEF Lmna$^{-/-}$	Telomere	pre-lamin A transfection	220

Typically, 12-20 telomeres or centromeres are tracked per cell. When tracking the LacI operon, only a single locus is tracked in each cell.

Supplementary Table 3: Effect of lamin A mutants on chromatin diffusion properties.

	Plasmid	Volume scanned by telomeres [μm^3]	Mean α	Number of telomeres
1	Without plasmid expression	0.1	0.99	503
2	eGFP-pre-laminA	0.012	0.57	220
3	GFP-L85R-lamin A	0.042	0.61	543
4	GFP-N195K-lamin A	0.051	0.85	508
5	GFP-R482W-lamin A	0.034	0.67	779
6	GFP-L530P-lamin A	0.052	0.75	723

Diffusion properties of telomeres in Lmna$^{-/-}$ cells, cells expressing normal lamin A protein and different lamin A mutant proteins. Both the exponential coefficient α and the volume scanned by the telomeres during 925 sec are shown.

Supplementary Reference

1) Kepten, E., Bronshtein, I. & Garini, Y. Improved estimation of anomalous diffusion exponents in single-particle tracking experiments. *Phys. Rev. E* **87**, 052713 (2013).
